# Too Far to Care? Measuring Public Attention and Fear for Ebola Using Twitter

**DOI:** 10.2196/jmir.7219

**Published:** 2017-06-13

**Authors:** Liza GG van Lent, Hande Sungur, Florian A Kunneman, Bob van de Velde, Enny Das

**Affiliations:** ^1^ Centre for Language Studies Radboud University Nijmegen Netherlands; ^2^ Communication Sciences University of Amsterdam Amsterdam Netherlands

**Keywords:** psychological theory, epidemics, fear, distance perception, social media

## Abstract

**Background:**

In 2014, the world was startled by a sudden outbreak of Ebola. Although Ebola infections and deaths occurred almost exclusively in Guinea, Sierra Leone, and Liberia, few potential Western cases, in particular, caused a great stir among the public in Western countries.

**Objective:**

This study builds on the construal level theory to examine the relationship between psychological distance to an epidemic and public attention and sentiment expressed on Twitter. Whereas previous research has shown the potential of social media to assess real-time public opinion and sentiment, generalizable insights that further the theory development lack.

**Methods:**

Epidemiological data (number of Ebola infections and fatalities) and media data (tweet volume and key events reported in the media) were collected for the 2014 Ebola outbreak, and Twitter content from the Netherlands was coded for (1) expressions of fear for self or fear for others and (2) psychological distance of the outbreak to the tweet source. Longitudinal relations were compared using vector error correction model (VECM) methodology.

**Results:**

Analyses based on 4500 tweets revealed that increases in public attention to Ebola co-occurred with severe world events related to the epidemic, but not all severe events evoked fear. As hypothesized, Web-based public attention and expressions of fear responded mainly to the psychological distance of the epidemic. A chi-square test showed a significant positive relation between proximity and fear: χ^2^_2_=103.2 (*P*<.001). Public attention and fear for self in the Netherlands showed peaks when Ebola became spatially closer by crossing the Mediterranean Sea and Atlantic Ocean. Fear for others was mostly predicted by the social distance to the affected parties.

**Conclusions:**

Spatial and social distance are important predictors of public attention to worldwide crisis such as epidemics. These factors need to be taken into account when communicating about human tragedies.

## Introduction

### General Background

In early spring of 2014, the world was startled by an outbreak of Ebola: a fairly unfamiliar and incurable virus with a high risk of death. Starting in March in Guinea, the epidemic quickly spread to West African cities, causing over 10,000 infections and 5000 deaths in a course of 9 months [1]. Although Western media covered the main events relating to the unfolding Ebola crisis in West Africa, especially psychologically close events appeared to cause a stir in the West. For instance, the reporting of a few Ebola cases in the United States led to demonstrations in favor of a travel ban even for countries like Saudi Arabia, where Ebola did not actually prevail [2], and in Western Europe significant public attention was paid to the euthanasia of a dog that belonged to an infected Spanish nurse [3].

When do humans start paying attention to real-world events? What aspects of an epidemic are most likely to increase fear? How can one adequately isolate the factors responsible for sudden surges in public attention and fear following health crises like the Ebola outbreak? The present research uses Twitter to examine the role of real-time changes in spatial and social distance to the epidemic to understand shifts in public attention and fear during a crisis. Previous research used Twitter as a proxy for disease activity [4-6] and to assess real-time public sentiment and opinion [7-10]. The present research complements these findings by applying construal level theory (CLT) [11] to examine Web-based attention and fear in public crises. The present research also contributes to CLT literature by being one of the few studies to examine CLT assertions beyond experimental laboratory conditions with longitudinal data [12,13].

### Construal Level Theory of Psychological Distance

CLT is a psychological theory that explains the relationship between psychological distance of stimuli (eg, events, objects, and people) and how they are mentally represented or construed [11,14]. Psychological distance refers to the subjective distance stimuli maintain from a person’s direct experience [15], which is centered around “here,” “now,” the “self,” and “reality.” Based on CLT, as the Ebola epidemic comes closer on these four dimensions (ie, spatially, temporally, socially, or hypothetically), it also becomes “psychologically closer.” According to CLT, psychological distance plays a fundamental role in shaping mental representations [14,16]. Whereas psychologically close stimuli tend to be represented in a detailed, contextualized, and concrete manner (ie, low-level construal), psychologically far stimuli are represented more generally and abstractly (ie, high-level construal) [14,17]. The association between psychological distance and construal level has been successfully applied to explain various psychological processes such as biases and decision making [12,14], and the CLT framework has been increasingly applied by communication scholars [18-21].

Psychological distance has important implications for risk perceptions and experiencing affect. Previous research has shown that fear and arousal for (real or imagined) negative events decrease with increased psychological distances [22,23]. Similarly, perceptions of risk for negative events, such as a health hazard, have shown to increase with psychologically close framing [24], whereas increased psychological distance is linked to lower risk perceptions [13]. Low-level construals (and close psychological distances) have also been linked to increased truth [25,26] and likelihood perceptions [15,27,28] (eg, risk of contracting a disease), and higher behavioral intentions [29] compared with high-level construals (and far psychological distances). The underlying reason for these effects is suggested to be the more concrete and detailed nature of mental representations in closer psychological distances. The reduced intensity of affect caused by high psychological distance is also suggested to be due to the critical role physical distance has for human biology and survival [22]. In line with these findings, a previous study showed that as time passed, people used less anxiety- and sadness-related words on Twitter about the Sandy Hook Elementary School shooting [30]. However, the same study also showed that an increase in spatial distance caused a decrease in the number of sadness-related words but an increase in the number of anxiety-related words. In addition, “focusing on the abstract causes of this tragedy (rather than the concrete details) decreased sadness (...) but increased anxiety” (p.370). Although highly relevant to this research, the Sandy Hook shooting was one big impactful event in the United States. This research examines responses to an impactful health crisis as it unfolds across different continents over the course of several months.

### Psychological Distance, Severity, and Fear for Epidemics Expressed on Twitter

Previous social media findings suggest that although media patterns sometimes converge with epidemic curves [5], they appear better suited to track public opinion and sentiment. This is because the Web-based media patterns more often follow the agenda of classical news media about an epidemic in an area of focus [7,8]. Classical media curves often do not converge with epidemic curves but rather are governed by the laws of news values. For example, the first infection in an epidemic has higher news value than the 1000th infection [31]. Although the relationship between classical and Web-based responses has been examined within one specific region, little is known about the relationship between gradual changes in psychological distance to certain real world events (eg, increased infection rates) and public attention and sentiment. Gaining more insight into this relationship is important to obtain generalizable insights about human responses to health crises. Specifically, 2 key theoretical questions have not been answered by previous CLT studies: (1) “when do individuals start experiencing events as psychologically close?” and (2) “does the severity of a (psychologically far or close) event play a role in public sentiment?”

Building on previous research regarding the use of social media as a real-time public opinion and sentiment proxy, we examined Twitter patterns as the Ebola epidemic approached the area of focus, in this case the Netherlands. We connected these patterns to specific locations (eg, West Africa and Spain) mentioned in the Tweets in order to examine the relationship between psychological distance of the outbreak, public attention, and fear. Based on CLT, as psychological distance decreases, mental representations of the events concerning the Ebola outbreak should become more concrete. We therefore expect increased public attention, as expressed by the number of tweets, as the Ebola outbreak becomes psychologically closer (Hypothesis 1). An earlier Twitter study found that surveillance of flu infection rates could be improved by using tweets that contained reference to “self” and “infection” (vs “other” and “concerned awareness”) [4]. This gives reason to think that a similar difference could be observed for fear; people may not fear becoming infected with Ebola unless an epidemic becomes psychologically close. This implies that especially expressions of fear for self should respond to reports of increased psychological closeness of an epidemic (Hypothesis 2).

It also remains unclear to what extent the affective value of an event itself plays a role in the relationship between psychological distance and public sentiment. Journalism studies suggest that severity is a factor that crucially determines the prominence of a certain real-world event. For example, 1000 casualties have bigger news value than a small number of casualties and will consequently trigger higher levels of media attention [32]. However, media coverage of severe epidemics also includes key political and economic events such as political debates about the epidemic or travel bans [32]. As social media follows classical media patterns, the severity of health events may only partially direct public attention on Twitter, together with key (nonhealth) events related to the epidemic. Previous studies showed that media volume curves appear to align more with public opinion than the epidemic curves, but these studies mostly investigated this relationship in a rather static manner, as they occurred within the same region. As the vast majority of Ebola cases were observed in West Africa, a comparison of Twitter curves in the Netherlands with the epidemic curves and severe world events relating to the epidemic should provide new insight into the relationship between the severity of the epidemic, and public attention and fear in a more global and transient manner. [7]. We therefore examined the relationship between public attention for Ebola on Twitter on the one hand and infection and mortality rates on the other (RQ1).

## Methods

### Material

A corpus of 185,253 Dutch tweets containing “(#)Ebola” was built, dating from the first outbreak (and report) of Ebola on March 22, 2014 to October 31, 2014. We collected the tweets from the TwiNL archive, a corpus of Dutch tweet IDs posted from December 2010 onward [33]. The peaks of Ebola prevalence in the corpus were connected to corresponding real-world events in the news and to infection and mortality rates, as counted by the Centers for Disease Control and Prevention (2015). A sample of 4500 tweets was selected by using the randomize function of Microsoft Excel and was then hand-coded by 3 independent coders who each coded 2000 tweets.

### Manual Encoding

Tweets were encoded for “fear” as (1) fear for self, (2) fear for others, and (3) no fear. The coders used fear-related keywords and emoticons such as: fear, scare(d), threat, help, scary, brr, OMG (oh my god), WTF (what the fuck), danger(ous), panic, dare (not), creepy, infected, contagious, death(s), care, symptoms, hospital, ill(ness), die(d), mortal(ity (rate)), spread, infection (rate), risk (group), :(, :’(, :O [8,34]. Whether these were indeed designators of fear (and not just of risk) was evaluated in the context of each tweet. Furthermore, simple notifications of reported fear without emotional connotations were coded as “no fear,” even when they contained the expressed worries from others. The difference between fear for self and others was based on the use of subjects, verbs, and (personal) pronouns [4]. When the (implied) subject was “I” or “we,” that tweet was encoded as “fear for self” and otherwise as “fear for others.” The intercoder reliability was high (Cohen kappa=.930).

To assess the “psychological distance” of Ebola toward the Netherlands, tweets were coded into one of the twelve different categories: (1) the Netherlands, (2) neighboring countries, (3) West and North Europe, (4) South Europe, (5) East Europe, (6) North America, (7) North Africa, (8) West Africa (Guinea, Sierra Leone, Liberia), (9) South Africa, (10) South America, (11) Asia and Oceania, and (12) no location. Tweets were assigned a specific “distance” category when the tweet explicitly expressed a location within the concerning area (eg, when a tweet mentioned a potential case in Zurich, that tweet was encoded as “(3) West and North Europe”). When a tweet did not mention a specific location, it was encoded as “(12) no location.” When a tweet mentioned multiple areas, the code of the nearest area was granted.

To our knowledge, no previous research identified the psychologically close or far countries or areas for the Dutch population. Therefore, this classification was based on the dimensions of psychological distance proposed by CLT. Following considerations of spatial and social distance, we formed the categories by starting from the areas the Dutch people presumably regard the nearest and continued categorizing toward the furthest areas. According to CLT, people form higher number of specific categories for low and fewer or broader categories for high psychological distances [35]. We therefore categorized a higher number of more specifically formed groups within close spatial distance of the Netherlands (ie, Europe is divided in groups 1 to 5), and broader groups out of areas with high spatial distance (eg, Asia and Oceania form group 11). North America is classified as the sixth nearest group; although it was not the next spatially closest area, we reasoned it will probably be seen as psychologically closer due to the closer social distance it implies for the Netherlands. West Africa was classified separately because it was thematically relevant as the Ebola epidemic originated there. When a tweet mentioned multiple areas, the code of the nearest area was applied. Intercoder reliability was high (Cohen kappa=.911).

### Time Series Analysis

Relations between time series were estimated and corrected using vector error correction models (VECMs) to account for cointegration (ie, tendencies to equilibrium) in different time series with lags based on empirical results using Aikake information criterion. The model to support claims regarding fear was specified using the Johansen procedure for determining cointegration with alpha *=*.05 threshold. These series were logged and corrected for trends after determining the original series contained violations of the stationarity assumption using the Kwiatkowski et al unit root test with constant; violations of normality detected by the Jarque-Berra residual normality, skew, and kurtosis test; and remaining autocorrelation by using the multivariate Portmanteau- and Breusch-Godfrey test for serially correlated errors. The logged model showed no signs of serial correlations (χ^2^_224_*=* 234.4 *, P*>.05) or of lingering heteroscedasticity *,* although non-normality persisted. The new-deaths and new-cases series were used as exogenous dummy variables. The research question was modeled using the same tests. In this model, non-normality also persisted but no heteroscedasticity was observed. In both cases a visual inspection of the residual plots did not indicate strong biases relevant to the reported results. In addition, we tested robustness of findings with different imputed breakpoints identified through the Zivot and Andrews unit root test and saw no significant changes. The expressed correlations are based on the error-corrected vectors of the error correction model (ECM) time series.

## Results

### Social Media Curve Versus Epidemic Curve

**Figure 1:**
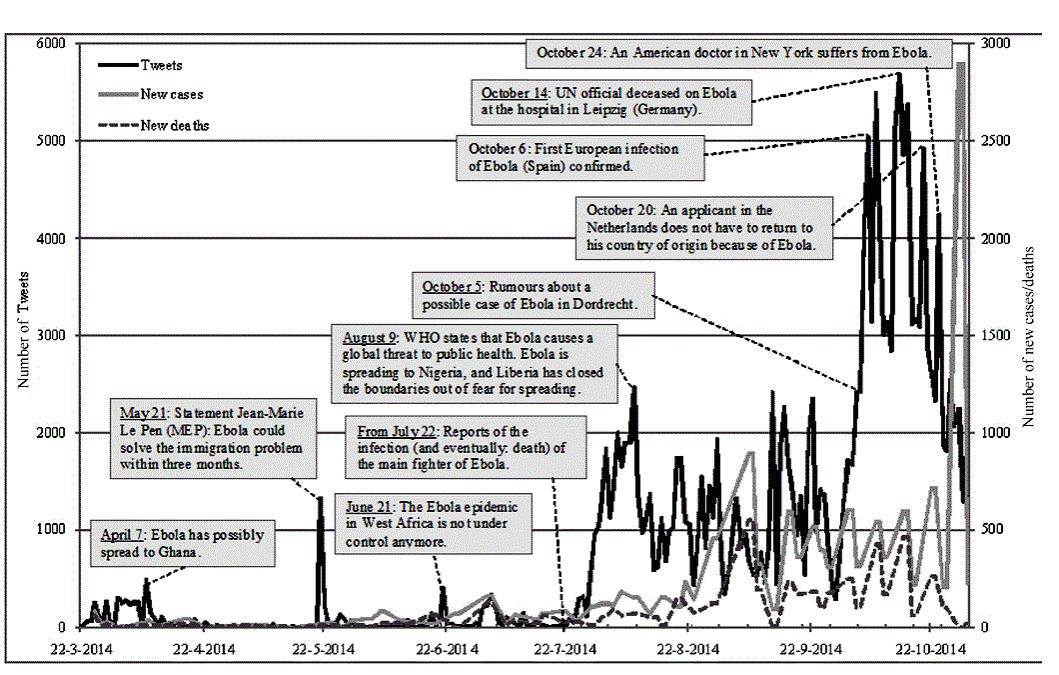
The daily Dutch tweet volume about Ebola from March 22 to October 31, 2014 (N=185,253); and the reported new cases (N=13,540) and deaths (N=4941) caused by Ebola in Guinea, Liberia, and Sierra Leone, according to the Centers for Disease Control and Prevention (2015). The primary bar lines indicate one month, the secondary one week.

**Figure 2:**
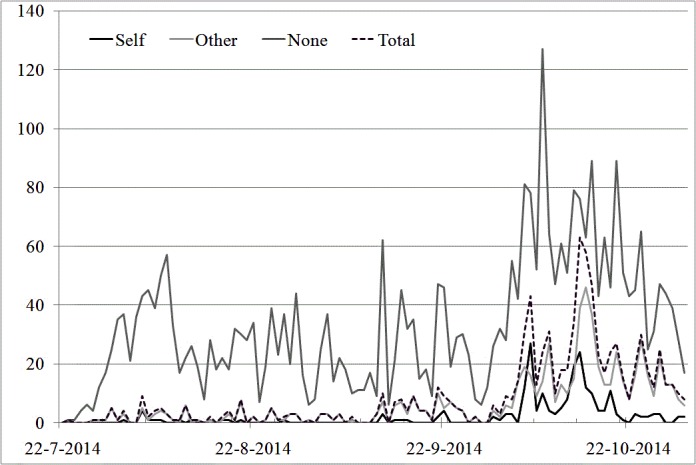
The daily amount of coded tweets containing fear for self or other or none plotted over time from July 22 to October 31, 2014. The primary bar lines indicate one month, the secondary one week.

**Figure 3:**
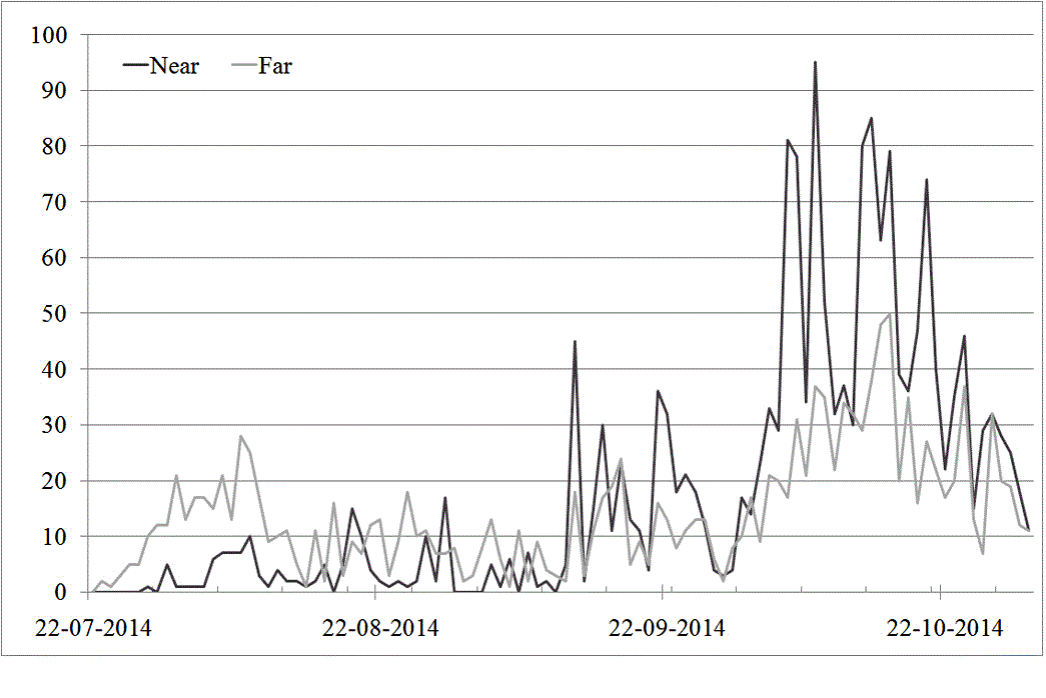
The daily amount of coded tweets about Ebola, related to near (Netherlands and neighboring countries) versus far (all other) locations, from July 22 to October 31, 2014. The primary bar lines indicate one month, the secondary one week.

Figure 1 shows that at the onset of the epidemic Ebola received fairly little Twitter attention in the Netherlands. From March 22 to July 21, 2014, 8600 tweets (4.64%) were sent, of which the most encoded tweets either did not contain a location (57.7%, n=105) or referred to West Africa (34.1%, n=62). Only 13 of those tweets contained fear (both for self and others). Figures 2 and 3 show data on July 22, 2014, after which an increase in the daily tweet volume can be observed. The Twitter curve does not coincide well with the epidemic curves for the number of Ebola cases and deaths, whereas the total cases and deaths grow very fast at the end of October, the tweet volume goes down. At first glance, the Twitter curve mainly coincides with real-world events in relatively psychologically close areas; for example, when Ebola crossed the Mediterranean Sea and the Atlantic Ocean (suggesting increased proximity), peaks in the tweet volume can be observed. A VECM (*r=* 2) causality analysis of differences confirmed that new reported infections and new reported deaths do not seem to granger cause (*F*_6663_=0.19, *P*>.05) or instantaneously cause (χ^2^_2_=0.3, *P*>.05) the number of tweets. This answers RQ1, showing that the epidemic curve and social media curve do not coincide.

### Ebola-Related Fear as Expressed in Dutch Tweets

Figure 2 shows the daily number of fear expressions (self, other, total, and none), plotted over time. About 19.9% (n=896) of the coded tweets contained fear for Ebola, but the majority (80.1%, n=3604) did not. The figure shows two fairly even trends for fear for self and others, wherein the frequency for self (4.9%, n=222) was lower than for others (15.0%, n=674) during almost the entire period. “Total” and “none” fear did not always follow the same pattern, and the number of occurrences for no fear was the highest during the entire period. Comparing the tweets coded for fear with the overall number of Ebola related tweets, it becomes clear that “no fear” has the best correlation with the total Twitter curve. After correcting for exogenous influences of the events on the ground and the cointegration of these time-series (Johansen procedure *r=2*), “no fear” shows strongest correlation with the overall level of tweets (*r*_222_*=*.95 *, P*<.05). “Self” (*r*_222_*=*.56 *, P*<0.05 *)* and “other” (*r*_222_*=*.73 *, P*<0.05 *)* show more modest associations. Fear for self and others follow a different pattern, especially in the early phase, with correlations before August 18, 2014 of *r=*.47 and *r=*.59 after cointegration correction respectively. The first significant peak of fear for others is not seen until October 5, 2014, and although “self” increases as well, its first peak is seen the next day. On those days, there were notifications of a potential (later disconfirmed) Ebola patient in the Dutch city Dordrecht (October 5, 2014), and the first confirmed spread to Europe (October 6, 2014). On October 13, 2014, a notification was sent about a potential Ebola patient in a Belgian hospital; this news event is followed by the second peak for “self” on October 14, 2014 and for “others” on October 15, 2014.

### Fear as a Function of Psychological Distance

As shown in Table 1, the percentage of fear for self was highest for the Netherlands (8.5%, n=134) and neighboring countries (18.5%, n=49). When a tweet did not specify a location, that tweet showed no fear for Ebola in almost all situations (90.1%, n=1053). Areas observed less than 100 times in total were excluded as separate values because of their possibly distorted percentages, but they were included in the “total” percentages. For the Netherlands and West Africa, percentages of tweets displaying fear for others amounted to around 16%; for the other locations (save “no location”), these percentages amounted to around 20-25%. The fairly low percentage for West Africa is particularly striking given the vast majority of infections and deadly cases reported in this area, compared with only few incidental infections in other regions.

**Table 1:** Location versus fear: crosstab of the percentage tweets per location (N&gt;100) that contained fear for self, fear for others, or no fear. The excluded locations (N&lt;100) are taken into account in the “total” percentages.

Location	Fear		
	Self	Others	None
Netherlands (n=1572)	8.5	16.2	75.3
Neighboring countries (n=265)	18.5	22.6	58.9
South Europe (n=113)	1.7	20.4	77.9
North America (n=362)	0.6	24.3	75.1
West Africa (n=632)	1.1	15.4	83.5
No location (n=1174)	1.5	8.5	90.0
Total (N=4500)	4.9	15.0	80.1

Nevertheless, it remains difficult to draw final conclusions about a possible correlation between fear and psychological distance. Therefore, a new proximity variable was created by merging the Netherlands and neighboring countries under the category of “near” and all other locations under “far.” “No location” tweets were regarded missing, as they did not indicate distance (see Table 2). A chi-square test showed a significant positive relation between proximity and fear: χ^2^_2_=103.2 (*P*<.001). Table 2 suggests that fear for self was found significantly more often than expected when linked to near locations, and significantly less when linked to far locations. The frequencies for fear for others and no fear did not differ significantly. Figure 3 further shows that the number of tweets about near locations increased particularly in the beginning of October, when the first Ebola cases were reported in Europe. Tweets on far locations are much less frequent during this time frame, in spite of significant increases in infections and deaths reported in West Africa (Figure 1). These findings confirm H1 and H2, that proposed that public attention and fear for Ebola decreased as a function of psychological distance.

**Table 2:** Psychological distance versus fear: crosstab for the percentage of tweets containing fear for self, fear for others, or no fear when location is near or far from the Netherlands, with the standardized residuals in brackets.

Psychological distance	Fear		
	Self	Others	None
Near (n=1837)	9.96 (6.5)	17.09 (−0.5)	72.95 (−1.6)
Far (n=1493)	1.28 (−7.4)	18.23 (0.6)	80.48 (1.8)
Total (N=3330)	6.20	17.59	76.21

## Discussion

### Principal Findings

This research on social media was, as far as we know, the first to examine the role of psychological distance in real-time Web-based responses to an approaching epidemic. Whereas previous studies have examined how social media relates to epidemic curves and classical media, the influence of psychological distance and severity of key events had not been explored. Current findings suggest that CLT may be a useful framework to increase understanding of public response to epidemic outbreaks. Even though the vast majority of Ebola cases occurred in West Africa and only a few suspected cases appeared near the Netherlands, findings showed that public attention for Ebola did not coincide well with the epidemic curve. As hypothesized, public attention for Ebola and expressions of fear for Ebola mostly responded to psychologically close events. Especially “fear for self” responded to increases in psychological closeness of the Ebola outbreak. Overall, findings suggest that events occurring in psychologically far regions of the world do not automatically capture public attention, even if the events are very severe.

The findings extend previous CLT findings in several ways. First, whereas previous research showed the effects of psychological distance in offline contexts [16,26,28], this has not been fully investigated in relation to Web-based communication contexts. It has been suggested that Web-based environments permit more confounded relationships for psychological distance dimensions (eg, with videoconferencing one can interact with spatially distant others in real time) compared with offline contexts [36]. Whether spatial distance is still important in Web-based contexts is a debated topic [37-39]. Therefore, this research contributes to CLT literature by demonstrating influence of two dimensions of psychological distance; namely spatial and social distance in Web-based expressions of public attention and fear. Second, our findings complement several lines of research that employ CLT framework to influence psychological distance perceptions in order to change behavioral intentions regarding important events. For instance, in climate change and distant suffering literature, psychological distance is suggested to be a barrier to engagement and behavioral intentions to act [13,29,40,41]. Our findings lend evidence to the application of CLT framework, and more specifically, the potential of rendering a situation psychologically close in order to increase attention to it.

The higher percentage of tweets expressing fear for self for neighboring countries compared with the Netherlands can be explained by the few confirmed cases of Ebola reported in, for example, Leipzig (Germany). Since both areas can be regarded as psychologically close in this study, following CLT we can reason that events in these areas were represented concretely [11], and perceived as truer [25] and more memorable [42] when compared with events taking place in distant locations. Whereas no overall relationship was found with regard to fear for others, a striking difference was observed between tweets involving North America and West Africa. Tweets about Ebola in North America (socially closer to the Netherlands) had the highest percentage fear for others, whereas tweets about Ebola in West Africa (spatially closer) had the lowest, in spite of extreme differences in reported cases between these regions. This suggests that people perceive psychologically far risks especially as more relevant for socially closer others. Social distance in this context might be more informative than spatial distance. Whereas this was not explicitly tested in this study, we can reason that people may have more knowledge about socially close others (eg, North America compared with West Africa) and can therefore imagine their situation more concretely and feel fear for those others. Subsequently, the findings extend previous studies on social media and health by showing that psychological distance not only affects perceptions of objects and events [20,21,23] but also determines (Web-based) public attention for an event and real-time expressions of fear (for self). The responses of the Twitter users might not be representative for the entire Dutch population, but the Netherlands have nevertheless a relatively large number of Twitter users compared with other countries, providing an adequate indication of the actual opinion and sentiment in the Netherlands.

In this research, and in line with previous studies [8,9], increases in attention for Ebola cooccurred with some severe real-world events, such as the World Health Organization (WHO) reports that Ebola formed a global threat. Yet, we can add that not all severe events evoked fear; public attention and fear overall responded strongest to proximity. The fact that attention and fear for Ebola in the Netherlands reached its peak around the time that Ebola started crossing the Mediterranean Sea and Atlantic Ocean suggests that the crossing of psychological boundaries may trigger sudden—rather than gradual—changes in proximity perceptions. Although severity of events played only a minor role in this research, it may play a role in and across other (closer) epidemics. Attention for Ebola was relatively minor compared with outbreaks as H1N1 [9], most likely because Ebola did not reach the Netherlands during the examined time frame. Future research should examine other epidemics that actually reached the Netherlands. The severity of an Ebola infection which takes place in a location where one has limited knowledge may be harder to visualize than an epidemic taking place in own country and thus, lead to more abstract mental representations [11]. Further research can test this possibility by examining differences in language abstraction (ie, the use of abstract vs concrete language) as a function of distance and severity [25].

### Conclusions

Even though humans may care morally and rationally for the tragedies and suffering of others, psychological distance of events exerts important boundaries on what attracts people’s attention and triggers their emotions. These findings point to the limits of the human condition—limits that could be taken into account when communicating about human tragedies. The use of gripping concrete stories may be especially important to bring psychologically far news events to readers’ attention.

## Abbreviations

ECM: error correction model
VECM: vector error correction model
